# Letter to the Editor – Update from Ukraine: Rehabilitation and Research

**DOI:** 10.5195/ijt.2022.6535

**Published:** 2022-12-13

**Authors:** Kyrylo S. Malakhov

**Affiliations:** V.M. Glushkov Institute of Cybernetics of the National Academy of Sciences of Ukraine

**Keywords:** Hybrid e-rehabilitation, Internet of Medical Things (IoMT), Remote patient monitoring, Ukraine, Research, Telehealth, Telerehabilitation

## Abstract

This Letter to the Editor provides an update on the research from the Glushkov Institute of Cybernetics of the National Academy of Sciences of Ukraine. The Institute's clinical research team is currently rehabilitating the military personnel of the Defense Forces of Ukraine with combat stress reaction and post-traumatic stress disorder using hybrid e-rehabilitation methods and techniques. Current research in the field of digital health and IoMT in Ukraine is creating innovative information technology for computerized electrocardiography. The conduct of rehabilitation and research in an active war zone with safety concerns, limited resources for research, and intermittent loss of power and water is very challenging.

October 22, 2022

Dear Ellen R. Cohn, PhD:

We are grateful that the May 2022 Special Issue of the *International Journal of Telerehabilitation* featured research from the Glushkov Institute of Cybernetics of the National Academy of Sciences of Ukraine. We appreciate the ongoing interest of the telerehabilitation professional community, and so six months later, offer this update.

The Institute's clinical research team (Kyrylo Malakhov – MSc, Researcher; Tetiana Semykopna – PhD, MD, Physical Medicine and Rehabilitation (PM&R); Illya Chaikovsky – MD, PhD multiple, FRMS, PMESC, Lead Researcher; Vitalii Velychko – DSc, Lead Researcher; Oleksandr Shchurov – MSc, Junior Researcher, Front-End Developer; and Oleksandr Palagin – Scientific Supervisor, Academician of the National Academy of Sciences of Ukraine, DSc, Professor, Deputy Director, Head of Microprocessor Technology Lab) is currently rehabilitating the military personnel of the Defense Forces of Ukraine with combat stress reaction and post-traumatic stress disorder using hybrid e-rehabilitation methods and techniques (Palagin, et al., 2022). These include remote patient monitoring with IoMT (Internet of Medical Things) devices, such as ultra-miniature ECG devices. The monitoring is conducted outside of the clinician's office.

The team is also working to determine the physiological price (i.e., a decrease in functional reserve during activity) of the soldiers of the Defense Forces of Ukraine. The more significant the reduction of the functional reserve during activity, the higher the physiological price is paid. This is being done via miniature electrocardiographic hardware and software complexes.

Current research in the field of digital health and IoMT in Ukraine is creating innovative information technology for computerized electrocardiography. The main goal in creating this technology is to render subtle changes in the electrocardiographic signal (via IoMT devices, such as ultra-miniature ECG devices) that are invisible during routine analysis, more informative (Chaikovsky, 2020), To achieve this goal, the team created an original method and software for analyzing electrocardiograms and heart rate variability.

Progress has also been made on the conduct of an ECG examination outside the clinician's office using IoMT devices and the representation of the resulting analysis in a visual form that is understandable not only to specialists.

The conduct of rehabilitation and research in an active war zone with safety concerns, limited resources for research, and intermittent loss of power and water is very challenging. Despite the hardships of war, our dedicated team is actively and persistently working together in undisclosed locations.

Sincerely,

Kyrylo Malakhov^1^ (On behalf of the clinical research team)

**Figure:**
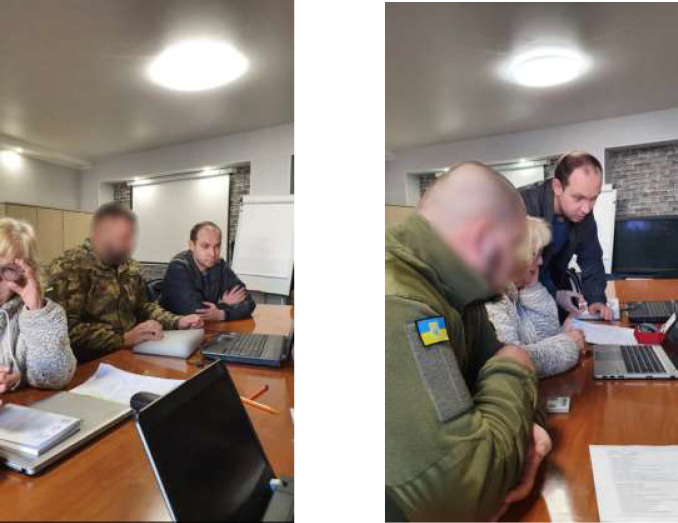


## Footnotes

1 Editor's note: The unblurred man in the photos is the letter's author, Kyrylo Malakhov. The unblurred woman in the photos is the research team member, Tetiana Semykopna.
